# Amino Acid Metabolic Enzymes in Gastric Cancer: Roles and Mechanisms in Tumorigenesis and Progression

**DOI:** 10.32604/or.2026.082561

**Published:** 2026-07-16

**Authors:** Zixin Wan, Jingdan Quan, Yue Qiu, Zhiwei Zhang

**Affiliations:** 1 Cancer Research Institute of Hengyang Medical College, University of South China, Hengyang, China; 2 Key Laboratory of Cancer Cellular and Molecular Pathology in Hunan Province, Hengyang, China

**Keywords:** Gastric cancer (GC), arginine, tryptophan, branched-chain amino acids, glutamine, metabolic enzyme, tumor microenvironment

## Abstract

Gastric cancer (GC) is one of the malignant tumors with high incidence and mortality worldwide. It has concealed early symptoms, poor prognosis for advanced patients, and limited efficacy of conventional treatments. Metabolic reprogramming is a core hallmark of cancer, among which amino acid metabolic reprogramming plays a critical regulatory role in the initiation and progression of GC. By linking intracellular energy supply, biosynthetic demands, and tumor microenvironment remodeling, it participates in immune escape, redox homeostasis maintenance, and therapeutic resistance. Dysregulation of key amino acids, including arginine, tryptophan, glutamine, branched-chain amino acids, serine/glycine, and aspartic acid, as well as altered expression and activity of rate-limiting enzymes and key catalytic enzymes, collectively drive the proliferation, invasion, metastasis, and stemness maintenance of GC cells. These metabolic enzymes can serve as potential biomarkers for the diagnosis and prognosis of GC, and are also important targets for precision therapy. At present, progress has been made in the development of inhibitors targeting key enzymes in amino acid metabolism. Single-target therapy or its combination with chemotherapy and immunotherapy has shown promising application prospects, but challenges such as clinical translation bottlenecks and unclear drug resistance mechanisms still exist. This review systematically summarizes the roles and molecular mechanisms of key amino acid metabolic enzymes in the occurrence and development of GC, and sorts out the research status of related targeted therapies, so as to provide references for basic research and clinical translation of metabolic precision therapy for GC.

## 1 Introduction

### 1.1 Epidemiology and Clinical Challenges of Gastric Cancer (GC)

GC ranks fifth in terms of both mortality and incidence rates among all cancers. Nearly a million new cases are diagnosed annually, leading to more than 650,000 fatalities globally [1]. GC development is influenced by multiple factors, including smoking, Helicobacter pylori infection, gender, age, race, and genetic predisposition [2]. Among these, *H. pylori* infection is the most recognized risk factor for distal GC [3]. Given its rather elusive initial symptoms, GC frequently gets diagnosed when it’s already in an advanced stage. The 5-year survival rate and the prospects of treating advanced GC remain poor despite growing knowledge of this illness and the creation of new medications [4], underscoring the pressing requirement for better screening to enable early detection and treatment. When it comes to treating GC, the available options encompass surgery, neoadjuvant radiotherapy, and chemotherapy. For early-stage cancers, surgical excision is still the sole effective treatment; nevertheless, the recurrence rate is substantial. In cases of advanced metastatic disease, healthcare providers may resort to standard chemotherapy, perioperative chemotherapy, or palliative chemotherapy. Nonetheless, the outlook is bleak, with under 5% of individuals with metastatic, advanced, or incurable GC surviving for more than five years [5]. While rates of intestinal-type GC have declined in recent years due to improved sanitation, dietary changes, and greater awareness of the link between *Helicobacter pylori* contamination and the disease, the development of new therapies remains a pressing need.

### 1.2 Metabolic Reprogramming in GC

Metabolic reprogramming has been recognized as a core hallmark of cancer, supporting sustained proliferation, survival under stress, and immune evasion [6]. In GC, amino acid metabolism functions as a central regulatory hub, linking intracellular energy supply and biosynthetic demands with the remodeling of the extracellular tumor microenvironment (TME) [7]. Metabolic enzymes shape immune phenotypes by consuming or producing key metabolites; for example, by depleting essential amino acids (such as tryptophan and arginine) or accumulating immunosuppressive metabolites (such as kynurenine), thereby inhibiting anti-tumor immunity, recruiting immunosuppressive cells, and promoting tumor progression [8]. This framework establishes a systematic connection between metabolic enzymes, TME remodeling, and therapeutic resistance. Recent research shows a close link among tumorigenesis, progression, and metabolic reprogramming [9]. In this context, amino acid metabolism holds significant clinical relevance, correlating with diagnosis, prognosis, and treatment response [10]. Consequently, targeting amino acid metabolism shows considerable promise for enhancing cancer therapy [11].

Metabolic dysregulation of key amino acids—including arginine, tryptophan, glutamine, branched-chain amino acids (BCAAs), serine, glycine, and aspartate—constitutes a critical feature of amino acid metabolic reprogramming in GC.

Systemic metabolic reprogramming is ultimately implemented by specific rate-limiting enzymes [12]. Pathway-level alterations are directly reflected in the expression and activity of key catalytic enzymes, which serve as functional nodes that control metabolic flux, signaling output, and therapeutic response [9,10]. In the subsequent analyses, we will dissect each amino acid metabolic pathway by focusing on these core enzyme nodes. This review summarizes these aspects, aiming to clarify the regulatory role of cores enzyme nodes in GC amino acid metabolism and establish the association between systemic metabolic reprogramming and underlying molecular mechanisms.

**Figure 1:**
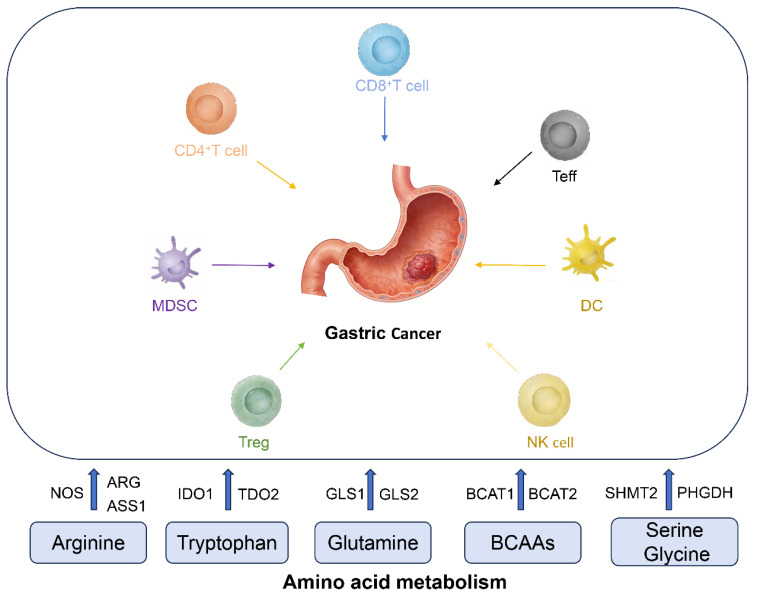
**Diagrammatic representation of the effects of amino acid metabolism on Gastric cancer (GC) tumor microenvironment (TME).** Immune cells in TME are impacted by amino acid metabolism, which results in tumor immune escape and aberrant immune factor and cytokine action. Abb: CD, Cluster of Differentiation; DC, Dendritic Cell; NK, Natural Killer Cell; MDSC, Myeloid-Derived Suppressor Cell; NOS, Nitric Oxide Synthase; ARG, Arginase; ASS1, Argininosuccinate Synthetase 1; IDO, Indoleamine 2,3-Dioxygenase; TDO, Tryptophan 2,3-Dioxygenase; GLS, Glutaminase; BCAT, Branched-Chain Amino Acid Transaminase; SHMT, Serine Hydroxymethyltransferase; PHGDH, Phosphoglycerate Dehydrogenase; BCAA, Branched-Chain Amino Acid.

It is noteworthy that amino acid metabolism does not function in isolation, but operates within the specific conditions defined by glycolysis and lipid metabolism [13]. In GC, under conditions such as nutrient stress, hypoxia, or epithelial-mesenchymal transition (EMT), amino acid dependency becomes significantly enhanced, as glycolytic flux can no longer meet the proliferative demands of tumor cells [14]. Unlike glucose metabolism, which primarily functions to supply energy, amino acid metabolism in GC is more focused on maintaining redox homeostasis, providing one-carbon units, mediating epigenetic modifications, and participating in immune suppression [15]. This context-dependent metabolic dominance explains why targeting amino acid metabolism, in combination with chemotherapy or immunotherapy, produces synergistic effects. Consequently, amino acid metabolism is positioned in GC as a compensatory and regulatory hub, capable of integrating the functions of multiple metabolic axes. (Fig. 1).

## 2 Amino Acid Metabolism in GC

### 2.1 Arginine

#### 2.1.1 Arginine Metabolic Pathways

A conditionally necessary amino acid, arginine is a crucial regulator of 80 different cellular physiological functions [16]. Research has established that dysregulation of the L-arginine (ARG) metabolic axis is fundamentally implicated in tumor development and progression [17]. Targeted modulation of arginine metabolic enzymes (e.g., nitric oxide synthase (NOS), argininosuccinate synthetase 1 (ASS1), arginase, and arginine decarboxylase (ADC)) or inhibition of cationic amino acid transporter (CAT) transporter-mediated arginine uptake represents a promising therapeutic strategy to suppress the development of tumors [16]. With its molecular mechanisms, including the coordinated interaction of several enzymatic processes and transporter systems, the regulation of arginine metabolism has recently become a key area of research in cancer therapies [18].

#### 2.1.2 NOS and Tumor Metabolism

L-arginine is broken down by NOS to yield nitric oxide (NO) and L-ornithine [19]. Numerous physiological processes are mediated by NO, a tiny free radical-related molecule. In physiological contexts, NO and its derivatives play a dual role in tumor growth, which is contingent upon their concentration [20]. Low doses of NO encourage the growth of cancer cells, inhibit programmed cell death, and boost processes including angiogenesis and metastasis by triggering several signaling systems, including the ERK, AKT, and mTOR ones, that are essential for tumor proliferation. In tumors, NO and its derivatives exert concentration-dependent dual effects on growth: low doses promote cancer cell proliferation, inhibit apoptosis, and enhance angiogenesis and metastasis by activating survival pathways (e.g., ERK, AKT, mTOR); high doses induce DNA damage, activate oncogenes, and promote tumor progression and metastasis [21].

#### 2.1.3 Arginase 1 (ARG1) and Tumor Metabolism

**Figure 2:**
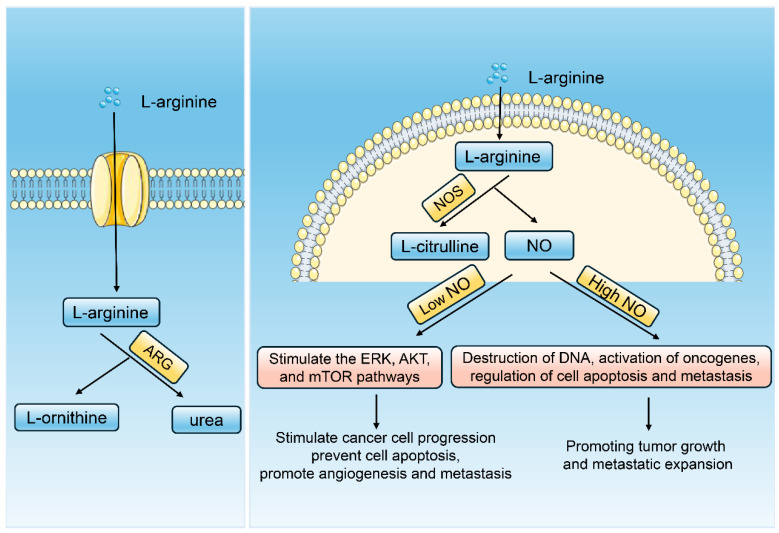
**Schematic of L-arginine metabolism in cancer cells.** L-arginine is catabolized by ARG into L-ornithine and urea, or metabolized by NOS to produce NO. Low and high concentrations of NO exert distinct pro-tumorigenic effects via different signaling pathways. Abb: NO, Nitric Oxide; NOS, Nitric Oxide Synthase; ERK, Extracellular Signal-Regulated Kinase; AKT, Protein Kinase B; mTOR, Mammalian Target of Rapamycin.

The human body contains two different forms of arginase: ARG1 and arginase 2 (ARG2) [22]. Recent studies have shown that ARG1 is upregulated in GC tissues, and its elevated expression leads to arginine depletion in the TME, thereby promoting GC cell proliferation [23]. Through the activation of myeloid-derived suppressor cells (MDSCs), which are immunosuppressive, ARG1 can alter cancer immune evasion [24]. Notably, emerging immunotherapeutic strategies are now directly targeting ARG1-expressing Tumor-Associated Macrophages (TAMs). For example, adoptive transfer of ARG1-specific CD4^+^ T cells can specifically recognize and reprogram ARG1^+^ TAMs, reducing the expression of ARG1 and other immunosuppressive markers such as Trem2, while promoting their repolarization toward a pro-inflammatory, M1-like phenotype. This targeted modulation remodels the TME, enhances antitumor immunity, and acts synergistically with immune checkpoint blockade therapy [25]. Ren and colleagues discovered a significant presence. The study explored the presence of tumor-infiltrating MDSCs in patients suffering from GC. They also observed that when the illness was first getting started, MDSCs were more likely to accumulate in the peripheral blood of these individuals, especially in those showing increased ARG1 expression levels [26]. (Fig. 2).

#### 2.1.4 ASS1 and Tumor Metabolism

ASS1 is the enzyme that regulates the rate at which arginine is produced. ASS1 expression and arginine availability are key metabolic determinants that influence tumor fitness and regulate immune interactions within the TME [27]. Numerous malignancies, such as ovarian, thyroid, pancreatic, colorectal, gastric, esophageal, cervical, and squamous cell lung carcinomas, have been found to have elevated expression levels of ASS1, which are linked to lower survival rates [28,29]. In situations where glucose is scarce, research shows that increased levels of ASS1 contribute to the survival of cancer cells, both *in vitro* and *in vivo*. This occurs by enhancing the nitrosylation of enzymes such as pyruvate carboxylase and phosphoenolpyruvate carboxykinase 2. As a result, the truncated gluconeogenesis pathway is facilitated, leading to an uptick in the production of serine, glycine, and purines. It is this rise in NO that ultimately aids in the endurance of cancer cells [28]. ASS1 can also promote GC metastasis by causing increased aggressiveness, suggesting it may serve as a tumor marker that can forecast GC patients’ survival and metastases [30].

### 2.2 Tryptophan

#### 2.2.1 Tryptophan Metabolic Pathways

**Figure 3:**
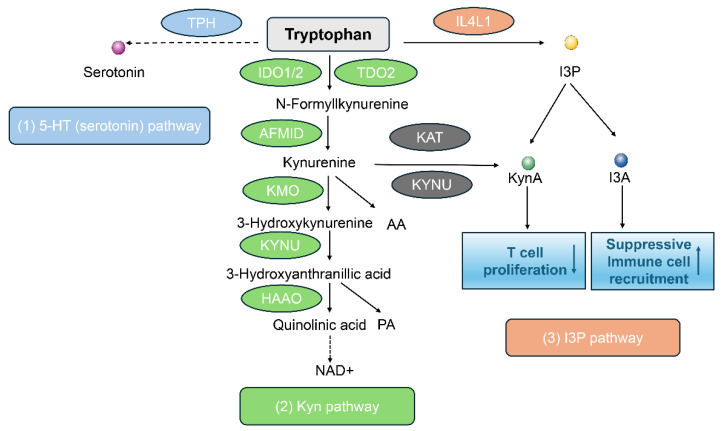
**Schematic of tryptophan metabolism pathways.** Tryptophan is metabolized via three major routes: the 5-HT (serotonin) pathway, the kynurenine (Kyn) pathway (the primary catabolic route generating NAD+), and the I3P pathway, which regulates immune cell function and promotes an immunosuppressive microenvironment. Abb: TPH, Tryptophan Hydroxylase; AFMID, Arylformamidase; KAT, Kynurenine Aminotransferase; KMO, Kynurenine 3-Monooxygenase; KYNU, Kynureninase; HAAO, 3-Hydroxyanthranilic Acid Oxygenase.

One necessary amino acid that limits the rate is tryptophan [31]. The three main tryptophan metabolic pathways utilized by inflamed organs and tumors are as follows: (1) The kynurenine (Kyn) route, which includes tryptophan 2,3-dioxygenase 2 (TDO2), indoleamine 2,3-dioxygenase 1 (IDO1), and IDO2. (2) Tryptophan hydroxylase 1 (TPH1) is a component of the serotonin (5-hydroxytryptamine: 5-HT) synthesis pathway. (3) The interleukin-4-induced gene 1 (IL4I1)-related route for the synthesis of indole-3-pyruvate (I3P) and its derivatives [32,33]. (Fig. 3).

#### 2.2.2 Tryptophan-Kynurenine-Aryl Hydrocarbon Receptor (Trp-Kyn-AhR) Pathway and Immunosuppression

In GC, the immunosuppressive effect of tryptophan metabolism is mainly mediated by the Kyn pathway, which is a key mechanism of tumor immune escape [34]. More than 95% of intracellular tryptophan in tumor cells is degraded via this pathway [35]. The activities of the key rate-limiting enzymes IDO1 and/or TDO2 are frequently significantly upregulated in GC, leading to tryptophan depletion and kynurenine accumulation in the tumor microenvironment [36]. Tryptophan deficiency activates the general control nonderepressible 2 (GCN2) kinase in T cells, suppressing their proliferation and function. Accumulated kynurenine, acting as an endogenous ligand for the AhR, directly triggers AhR nuclear translocation. Activated AhR induces the differentiation and functional enhancement of regulatory T cells (Tregs) by regulating downstream target genes such as IL-10 and TGF-β, while inhibiting the function of effector T cells and natural killer (NK) cells, thereby establishing a profoundly immunosuppressive TME [37,38]. Studies have shown that high IDO1 expression in GC tissues correlates with poor prognosis and immune cell infiltration status [39], and it can further reinforce the immunosuppressive environment through signaling pathways including phosphatidylinositol 3-kinase/protein kinase B/mammalian target of rapamycin (PI3K/Akt/mTOR) [40]. Additionally, IDO1 regulates the differentiation of T lymphocytes via the PI3K/Akt/mTOR pathway, which in turn encourages the proliferation of stomach cancer cells [41].

#### 2.2.3 TDO2 and Tumor Metabolism

One of the primary rate-limiting enzymes in the initial stage of the metabolism of tryptophan, TDO2, has had its functions and mechanisms in GC gradually clarified by a number of studies [42]. According to other research, TDO2 development is strongly linked to the development of GC, the prognosis of patients, the degree of tumor immune infiltration, and the expression of programmed death-ligand 1 (PD-L1), a crucial immunosuppressive protein that tumor cells express. Furthermore, there is a positive correlation between the expression of CD44, a stem cell marker, and the level of TDO2 expression in GC tissue samples. Additional functional investigations have demonstrated that downregulating TDO2 expression not only prevents GC cells from proliferating, colonizing, and invading but also lowers the viability of GC organoids and the efficiency with which cell spheres form, indicating that TDO2 plays a key regulatory role in the malignant biological behaviors of GC cells [43].

From the perspective of molecular mechanisms, the upregulated expression of TDO2 promotes tryptophan degradation and generates more Kyn. Kynurenine can trigger the AhR signaling pathway because it is an endogenous agonist of the AhR. Once activated, AhR further affects oncogene expression, angiogenesis, cell survival, and immune cell function—all of which are closely associated with tumorigenesis and tumor progression. In GC, the upregulated expression of both AhR and TDO2 can synergistically activate the AhR signaling pathway, thereby further promoting the proliferation, invasion, and immune evasion of GC cells and driving the progression of GC [44].

Comprehensive analysis of the aforementioned clinical correlation and molecular mechanism studies reveals that TDO2 expression shows promise as a possible target gene for precision therapy of GC in addition to being an independent prognostic indicator for assessing patients with the disease. Specifically, targeting TDO2 to regulate the metabolic-signaling pathway it mediates might offer a fresh approach to treatment for halting the spread of GC.

#### 2.2.4 5-HT (Serotonin) Pathway

The 5-HT pathway metabolizes a tiny portion of tryptophan. Trp is converted to 5-hydroxytryptophan (5-HTP) by TPH1 or tryptophan hydroxylase 2 (TPH2) [40]. 5-HT affects cancer cells through multiple pathways, such as promoting cell proliferation by accelerating cell cycle progression, supporting autophagy, and inhibiting apoptosis. Moreover, it serves as an effective free radical scavenger, effectively neutralizing lipid peroxidation, which in turn markedly reduces ferroptosis and fosters the advancement of cancer. The PI3K/Akt/mTOR signaling pathway is impacted by the 5-HT receptor HTR2B in human gastric adenocarcinoma tissues, which increases the survival of GC cells. In addition to increasing the survivability of gastric adenocarcinoma cells under metabolic stress, this modulation inhibits ferroptosis and reduces cellular and lipid reactive oxygen species levels. Consequently, this process contributes to increased tumor growth and diminished survival rates for patients [45].

#### 2.2.5 I3P Pathway

According to research, by triggering the IL4I1-AhR signaling pathway, collagen extracellular matrix can help GC cells evade the immune system [46]. I3P, a metabolite generated from tryptophan, is essential for the formation of tumors [47]. IL4I1 inhibits ferroptosis by generating I3P from tryptophan, which directly scavenges free radicals and activates a program for antioxidant gene expression. This program triggers the expression of cytoprotective genes [48].

### 2.3 Glutamine (Gln)

#### 2.3.1 Glutamine Metabolic Pathways

**Figure 4:**
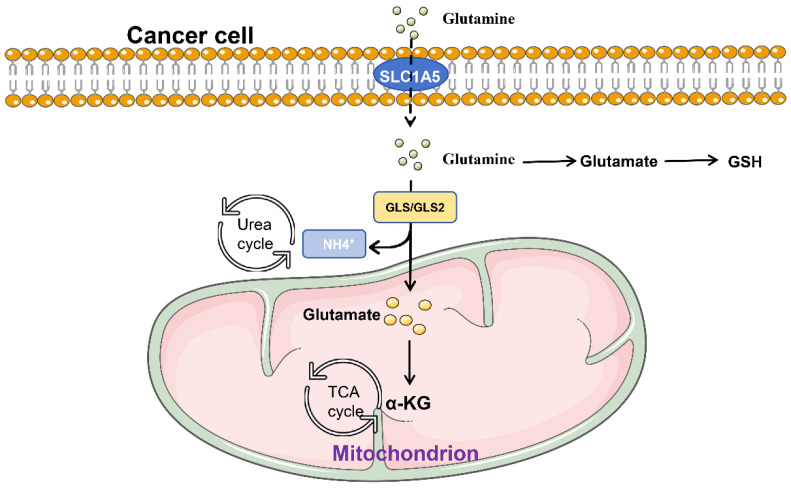
**Schematic of glutamine metabolism in cancer cells.** Extracellular glutamine is transported into cancer cells via the solute carrier family 1 member 5 (SLC1A5) transporter, and then catalyzed by glutaminase (GLS/GLS2) into glutamate. Glutamate is further metabolized to GSH for redox balance, or enters mitochondria to fuel the tricarboxylic acid (TCA) cycle through α-KG, while the byproduct NH_4_^+^ is cleared via the urea cycle. Abb: SLC1A5, Solute Carrier Family 1 Member 5; GLS, Glutaminase; TCA, Tricarboxylic Acid Cycle.

Despite being a non-essential amino acid, Gln can act as a conditionally essential amino acid under some situations [49]. Numerous biological processes, such as nucleotide synthesis, amino acid generation, protein glycosylation modification, extracellular matrix production, epigenetic modification, maintenance of cellular redox homeostasis, and autophagy, depend on glutamine metabolism to sustain tumor survival and progression [50]. Studies have found that Gln is a key driver of GC growth [51]. (Fig. 4).

Glutaminase (GLS) and glutamine synthetase, the two main enzymes involved in glutamine metabolism, catalyze the rate-limiting processes in glutamine production and use, respectively [52]. GLS stimulates the synthesis of glutamate and free ammonia. It is frequently elevated in multiple cancer types, facilitating tumor cell growth and preventing apoptosis [53]. Normal cells produce glutamine via GLS. In contrast, the glutamine made by tumor cells cannot meet their high demands for rapid growth, leading to a “glutamine-dependent phenomenon” [54].

Kidney-type glutaminase (GLS1) and liver-type glutaminase (GLS2) are the two main isoforms of GLS. GLS1 is linked to a poor prognosis and encourages carcinogenesis. Studies have found that glutamine provides an essential energy and material foundation for cancer cells [55].

#### 2.3.2 GLS1 and Tumor Metabolism

When GLS1 is downregulated or inhibited, glutamine’s conversion to glutamate and α-ketoglutarate (α-KG) encounters an obstacle that stops it from entering the tricarboxylic acid cycle. This problem disrupts the energy metabolism and biosynthesis of cancer cells, ultimately resulting in cell death and a cascade of biological repercussions. Many GLS1 inhibitors have demonstrated apoptotic effects on cancer cells. GLS1 is controlled by the oncogene c-MYC and has a positive relationship with tumor development [56,57]. High expression of GLS1 was detected in GC patients [55]. GLS1 activity inhibition can slow the growth of tumors and prevent their progression. Notably, GLS1 reshapes the tumor microenvironment by regulating glutamine metabolism: on the one hand, it consumes glutamine to cause nutritional deficiency, which inhibits immune cell function and promotes immune escape; on the other hand, it regulates epigenetics and oncogenic signals through metabolic products such as α-KG, thereby affecting genomic stability and drug sensitivity. As a core molecule connecting tumor metabolism, epigenetics, and immune suppression, GLS1 can serve as an important biomarker and target for tumor microenvironment targeting and immune combination therapy [58]. Consequently, GLS1 could serve as a valuable biomarker for the early identification and management of GC and might also function as a potential therapeutic target [59].

#### 2.3.3 GLS2 and Tumor Metabolism

GLS2 plays distinct roles in different tumor types, acting as either a tumor suppressor or an oncogene [60]. Research has shown that GLS2 demonstrates tumor-suppressing properties in tissues of GC. It was discovered that overexpression of GLS2 greatly reduced the migration and proliferation of GC cells and increased caspase 3 expression, which in turn promoted apoptosis [53]. Moreover, in hepatocellular carcinoma, GLS2 can curb cancer cell migration, invasion, and metastasis. It does so by mediating p53 function, negatively regulating PI3K/AKT signaling, and suppressing Rac1 activity [61]. GLS2 facilitates the conversion of glutamate to α-KG, which in turn increases the generation of reactive oxygen species (ROS) in lipids [62]. Ferroptosis, a type of iron-dependent cell death marked by the accumulation of lipid peroxides, is facilitated by this process. In the end, this mechanism prevents tumor cells from functioning [61].

Given that glutaminase is essential for tumor growth and serves as a crucial enzyme in the initial stage of glutamine breakdown, dual targeting of the two isozymes may become a key therapeutic tool for certain types of cancers and/or certain patients in personalized therapy [63].

### 2.4 Branched-Chain Amino Acids

#### 2.4.1 BCAAs Metabolic Pathways

The three primary essential amino acids—leucine, isoleucine, and valine—as well as vital nutrients and signaling molecules for cell growth and development, are known as BCAAs [64].

**Figure 5:**
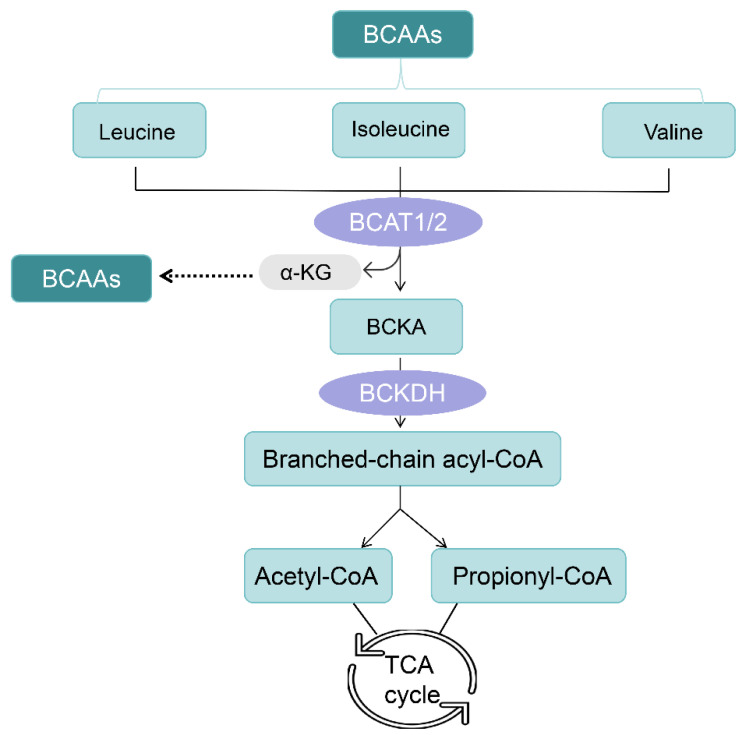
**Schematic of branched-chain amino acids (BCAAs) catabolic pathway.** BCAAs (leucine, isoleucine, valine) are first transaminated by BCAT1/2 to form branched-chain α-keto acids (BCKAs), with α-KG as a co-substrate that can be recycled back to BCAAs. BCKAs are then oxidatively decarboxylated by BCKDH to generate branched-chain acyl-CoA, which is further catabolized into acetyl-CoA and propionyl-CoA to fuel the TCA cycle. Abb: BCAT, Branched-Chain Amino Acid Transaminase; BCKA, Branched-Chain α-Keto Acids; BCKDH, Branched-Chain α-Keto Acid Dehydrogenase.

To create branched-chain α-keto acids (BCKA), BCAAs must first be transaminated by branched-chain amino acid transaminase1/2 (BCAT1/2). The same enzymes can change BCKA back into BCAAs. The alpha-keto acid dehydrogenase (BCKDH) complex then converts BCKA into branched-chain acyl-CoA. Following additional oxidation, branched-chain acyl-CoAs become acetyl-CoA and propionyl-CoA, which subsequently join the TCA [65]. (Fig. 5).

#### 2.4.2 BCAT1 and Tumor Metabolism

Research suggests a possible correlation between branched-chain amino acid (BCAA) catabolic enzymes and cancer [66]. BCAT is aberrantly expressed in numerous tumors. By triggering the Wnt/β-catenin signaling pathway and the PI3K/PKB/mTOR pathway, it frequently encourages tumor invasion and growth [67].

One study found that BCAT1 was overexpressed in GC tissues and positively correlated with the presence of TNM stage, local invasion, Lauren type, tumor classification, lymph node metastasis, and metastasis. By triggering the PI3K/AKT/mTOR pathway, BCAT1 may function as an oncogene by encouraging angiogenesis, invasion, and proliferation. Additionally, it stimulates vascular endothelial growth factor (VEGF) secretion, which raises *in vivo* concentrations of growth factors such as NO and angiopoietin [67]. The proto-oncogene c-Myc targets BCAT1 [68]. The potent transcription factor c-Myc affects apoptosis in a number of ways. While its maintenance prevents apoptosis, a reduction in c-Myc causes it [69].

#### 2.4.3 BCAT2 and Tumor Metabolism

Cancerous tumors express more BCAT2 than normal tissues in the majority of cancer types. However, BCAT2 is downregulated in GC tissues and is strongly associated with a poor prognosis [70]. According to research, BCAT2 may be a target for ferroptosis. BCAT2 affects intracellular redox equilibrium via controlling the metabolism of GSH and α-KG. Its upregulation increases GC cells’ resilience to drugs and their capacity to spread by suppressing ferroptosis, a type of iron-dependent lipid peroxidation-driven cell death. BCAT2 may be important in controlling the tumor immune microenvironment because it is strongly linked to the invasion of CD4^+^ T cells, CD8^+^ T cells, and regulatory Treg cells. These results show that BCAT2 is a promising biomarker for immunotherapy targeting and cancer prognosis [71].

### 2.5 Serine and Glycine

Both serine and glutamic acid are non-essential amino acids [72]. Serine and glycine are crucial for GC cell proliferation; their associated metabolic enzymes and metabolites regulate tumor growth [73].

Two main processes are involved in the synthesis of serine and glycine: the first is the *de novo* synthesis of serine from glucose, which diverges from glycolysis. After being oxidized to 3-phosphohydroxypyruvate (3-PHP) by phosphoglycerate dehydrogenase (PHGDH) [74], the glycolytic intermediate 3-phosphoglycerate (3-PG) is transaminated with glutamate as the amino donor to yield phosphoserine and α-KG, and phosphoserine phosphatase (PSPH) dephosphorylates it to yield serine. Second, serine hydroxymethyltransferase 1/2 (SHMT1/2) catalyzes the reversible interconversion of serine and glycine, which permits serine to be transformed into glycine and glycine to be reversibly transformed back into serine [75].

#### 2.5.1 PHGDH and Tumor Metabolism

PHGDH represents the first and rate-limiting enzyme in the *de novo* biosynthesis pathway of serine and glycine [76]. According to studies, PHGDH activity is the most consistently elevated in many malignancies and is strongly linked to tumor growth. It also plays a critical role in tumor spreading. PHGDH is a highly desirable target in the field of anticancer therapy development, as evidenced by studies showing that its expression levels in GC tissues are noticeably higher than those in benign or normal tissues [77]. Significant chemoresistance is a notable feature of GC of the serine metabolism-high (SEM) type. Research has revealed that these cancer cells exhibit resistance to the suppression of glutamine catabolism in addition to having a high expression of GLS. In the meantime, PHGDH’s catalytic activity and the overall effectiveness of the serine synthesis pathway are influenced by serine availability. Additional research has shown that SEM-type GC cells can sustain cell survival in the presence of glutamine starvation by upregulating the PHGDH-driven mitochondrial folate cycle pathway, which generates nicotinamide adenine dinucleotide phosphate (NADPH) to scavenge ROS [78].

#### 2.5.2 SHMT2 and Tumor Metabolism

The mitochondrial metabolic enzyme SHMT2 has been implicated in gastrointestinal malignancies in recent years. In order to produce the 1C units necessary for nucleotide biosynthesis, SHMT2 catalyzes the conversion of serine to glycine and transfers the hydroxymethyl group (Cβ) to tetrahydrofolate (THF). Moreover, SHMT2 is a key regulator of 1C metabolism in cancer cells that proliferate quickly [79]. In addition to being highly expressed in numerous cancer cells and boosting their ability to proliferate, SHMT2 has also been linked to esophageal, gastric, and colorectal cancers [80]. According to research, SHMT2 knockdown can stop GC from progressing in a number of ways, such as controlling the stability of the HIF1α protein, influencing the activity of the downstream VEGF and signal transducer and activator of transcription 3 (STAT3) signaling pathways, and preserving the redox homeostasis of GC cells in hypoxic environments [81].

### 2.6 Aspartic Acid

In GC cells, aspartic acid and asparagine play a dual role by facilitating cell proliferation and modulating intracellular signaling. Research indicates that supplementing with exogenous asparagine can sustain protein translation and encourage tumor proliferation in tumor cells facing glutamine scarcity. Conversely, reducing levels of aspartic acid has been found to impede tumor development [82,83].

## 3 Crosstalk between Amino Acid Metabolic Pathways in GC

Amino acid metabolic pathways do not function independently; they converge at shared metabolites, signaling nodes, and TME interactions to form a coordinated regulatory network in GC [84,85].

### 3.1 Shared Metabolic Intermediates

α-KG serves as a central bridge connecting glutamine, BCAA, and serine/glycine metabolism. It supports TCA anaplerosis, epigenetic modification, and ferroptosis resistance [86,87,88]. GSH links glutamine, BCAA, and tryptophan metabolism to maintain redox balance, which is critical for GC cell survival under oxidative stress [89].

### 3.2 Convergent Signaling Hubs

The mTORC1 and GCN2 pathways serve as universal sensors that integrate signals from all amino acid pathways. mTORC1 is activated by arginine, BCAA, and glutamine to promote protein synthesis and proliferation [90]. GCN2 triggers the integrated stress response (ISR) upon amino acid depletion, supporting GC adaptation to nutrient limitation. The PI3K/AKT axis is commonly activated by ARG1, BCAT1, IDO1, and GLS1, forming a convergent signal for GC growth and invasion [91,92].

## 4 Therapeutic Strategies and Challenges

### 4.1 Arginine Metabolism-Targeted Therapy

Targeting amino acid metabolism represents a highly promising therapeutic approach for GC, with evolving progress from basic research to clinical translation. Early studies mainly focused on single-target interventions. Arginine deprivation therapy using ADI-PEG20 and PEG-BCT-100, based on arginine auxotrophy, can directly kill tumor cells and enhance anti-tumor immunity, and dietary arginine restriction also shows potential value, although its definite efficacy in GC remains to be verified [30,93,94,95]. Notably, the dual roles of NO and the contradictory functions of ASS1 in different tumors pose considerable challenges to related research. As a rate-limiting enzyme in arginine biosynthesis, ASS1 exerts a consistent oncogenic role in GC [96], but acts as a tumor suppressor in other tumors [97,98]. This difference mainly stems from significant heterogeneity in cellular metabolic patterns, molecular regulatory pathways, and the TME between GC and tumors such as melanoma and sarcoma [99,100]. Therefore, ASS1 inhibition and arginine restriction are promising therapeutic strategies for suppressing metastatic tumor growth. We propose that ASS1 inhibition in GC confers a survival advantage to GC patients receiving pegylated arginine deiminase therapy. Further studies are needed to investigate the protein expression of ornithine carbamoyltransferase (OCT) and ASS1 in GC specimens and their impacts on arginine deprivation therapy.

### 4.2 Tryptophan Metabolism-Targeted Therapy

For tryptophan metabolism, early drug development was dominated by IDO1 inhibitors to reverse immunosuppression [101]. This study lacks analysis of cross-disease immune-metabolic characteristics, and many described immune effects are not GC-specific. Relevant studies have analyzed the gene expression of CD4^+^ T cells in various diseases through machine learning, providing a reference for judging whether the tryptophan metabolism pathway is a universal mechanism or a tumor-specific program, which is crucial for the interpretation of GC-related biomarkers [102]. Although preclinical data show that IDO1 inhibitors have certain potential, their clinical efficacy is limited, which is attributed to the non-enzymatic functions of IDO1 and the compensatory activation of TDO2. In recent years, research focus has shifted to multi-target regulation, such as the development of dual-target inhibitors, to break through the bottleneck of clinical transformation [103].

The combined application of tryptophan metabolism inhibitors with immune checkpoint inhibitors, chemotherapy, or radiotherapy has attracted extensive attention [104]. Clinical trials have shown that some patient subgroups have improved overall survival (OS) and progression-free survival (PFS), but the overall clinical benefit is limited, and the optimal timing and sequence of treatment need to be further optimized [105].

5-HT receptor antagonists are a potential therapeutic option for GC, showing broad-spectrum anti-tumor activity *in vitro* [106]. However, current research on GC is limited to *in vitro* models, lacking *in vivo* verification, and the synergistic mechanism with chemotherapeutic drugs such as cisplatin remains unclear, requiring further in-depth study.

Despite significant progress in tryptophan metabolism research, there are still knowledge gaps that hinder clinical translation. Future studies should focus on exploring drug resistance mechanisms, screening reliable biomarkers through multi-omics technology, and developing optimized combination therapy regimens to promote the clinical transformation of amino acid metabolism-targeted therapy and improve patient prognosis.

### 4.3 Glutamine Metabolism-Targeted Therapy

GLS, the rate-limiting enzyme in the glutamine catabolic pathway, is a viable therapeutic target for developing novel anticancer medications [50]. Two major families of GLS inhibitors, BPTES and 968, have been shown to exert anticancer effects. Clinical trials are currently underway to evaluate B-839, a BPTES-based allosteric GLS inhibitor with enhanced oral bioavailability and potent inhibitory activity [107]. This drug holds significant potential in cancer treatment. CB-839 can significantly reduce tumor volume in the GC patient-derived xenograft (PDX) model and enhance the anti-tumor activity of PD-1 inhibitors. Its mechanism is related to reducing the infiltration of MDSCs in the TME [59].

Although targeting glutamine metabolism is a promising approach for cancer treatment, several challenges remain in developing clinically effective drugs. Inhibiting glutamine metabolism may lead to unintended consequences, such as reduced antitumor immunity and increased metabolic flexibility or adaptability of cancer cells.

Notably, GLS1 and GLS2 exhibit opposing functions in GC: GLS1 acts as an oncogene, while GLS2 functions as a tumor suppressor. This functional paradox arises from differences in subcellular localization, p53 regulation, and ROS modulation [108,109]. The curcumin analog WZ35, a natural small-molecule derivative, could trigger intracellular ROS accumulation, block glycolysis progression, and regulate key signaling molecules including YAP and JNK. It further suppresses malignant biological behaviors and metabolic reprogramming in GC cells, presenting a novel therapeutic candidate for gastric cancer intervention [110].

### 4.4 BCAA Metabolism-Targeted Therapy

The BCAT1 and BCAT2 genes related to (BCAA metabolism exhibit unique expression characteristics in GC. Among them, high expression of BCAT1 can drive tumor cell proliferation, while low expression of BCAT2 weakens its tumor-suppressive function, and this expression difference is closely related to the metabolic heterogeneity of GC [111]. In the research on BCAA metabolism-targeted therapy, BCAT1 inhibitors are potential therapeutic agents, but current studies are still in the preclinical stage, lacking *in vivo* experimental verification, and the synergistic mechanism with chemotherapy and immunotherapy also needs further clarification.

### 4.5 Challenges and Clinical Translation of Amino Acid Metabolism in Gastric Cancer

Although significant progress has been made in amino acid metabolism research in GC, both technical bottlenecks and core scientific questions remain, hindering its translation into precise diagnosis and therapy. Existing studies confirm that metabolic reprogramming is a central hallmark of GC development, and dysregulated amino acid metabolism is closely associated with tumor progression and immune evasion [15].

The clinical translation of amino acid metabolism research in precision oncology is hampered by key technical bottlenecks: inadequate *in vivo* metabolic flux monitoring, challenges in resolving tumor metabolic heterogeneity, inefficiencies in multi-omics data integration, a lack of practical clinical technologies, and inherent limitations of current detection methods [112].

Addressing these challenges requires concerted technological innovation and advanced multi-omics integration to unlock the full potential of targeting amino acid metabolism in precision oncology.

## 5 Conclusion and Outlook

Currently, the research and development of targeted drugs for amino acid metabolism-based therapy have made certain progress, and the targeted drugs corresponding to different amino acid metabolic pathways and their research progress are shown in the following table: (Table 1).

**Table 1:** Amino acid metabolism-related enzymes and their targeted inhibitors in gastric cancer.

Name	Gene/Protein Expression	GC-Specific Function	Inhibitors	Clinical Development Stage	Reference
ARG1	upregulated	Depletes arginine in TME, activates MDSCs, promotes immune escape and GC proliferation	CB-1158 OATD-02 nor-NOHA Peg-rhArg1	SHIN1:Tool Compound/Probe; Others: Preclinical	[113,114,115]
ARG2	upregulated	Promotes arginine catabolism and GC growth	OATD-02 C0021158 nor-NOHA	All: Preclinical	[115,116]
ASS1	upregulated	Enhances GC survival under glucose starvation, promotes metastasis	C-01 MDLA ADI-PEG 20	SHIN1:Tool Compound/Probe; Others: Preclinical	[117,118]
IDO1	upregulated	Degrades tryptophan, produces Kyn, activates AhR, induces immune suppression	GDC-0919 Indoximod Epacadostat Roxyl-WL β-Lapachone	All: Preclinical	[119,120,121]
TDO2	upregulated	Drives Kyn-AhR signaling, promotes stemness, PD-L1 expression and GC invasion	optimized compound 21 LM10 680C91 DN604-TDOi	SHIN1:Tool Compound/Probe; Others: Preclinical	[122,123]
GLS1	upregulated	Promotes glutaminolysis, fuels TCA cycle, supports GC proliferation	BPTES CB-839 AC16 JHU-083	All: Preclinical	[124,125]
GLS2	downregulated	Tumor suppressor; induces ROS, promotes ferroptosis and apoptosis	Compound 968 AV-1	SHIN1:Tool Compound/Probe; Others: Preclinical	[104,126]
BCAT1	upregulated	Activates PI3K/AKT/mTOR, enhances angiogenesis and GC invasion r	BAY-069 Gabapentin BMS-582664	All: Preclinical	[127,128,129]
SHMT2	upregulated	Regulates HIF1α/VEGF/STAT3, supports nucleotide synthesis in hypoxia	Lometrexol SHIN1 AGF347 SHMT-IN-2	SHIN1:Tool Compound/Probe; Others: Preclinical	[130,131,132]
PHGDH	upregulated	Rate-limiting for serine synthesis; mediates chemoresistance and metabolic plasticity r	NCT-503 CBR-5884 Withangulatin A (WA) B12 D8 Salvianolic Acid C/Schizotenuin F Chicoric Acid	All: Preclinical	[133,134,135,136,137]

Note: Abb: TME, Tumor Microenvironment; MDSCs, Myeloid-Derived Suppressor Cells; AhR, Aryl Hydrocarbon Receptor; TCA cycle, Tricarboxylic Acid Cycle; PDX, Patient-Derived Xenograft; GC, Gastric Cancer; GLS, Glutaminase; BCAA, Branched-Chain Amino Acid; BCAT1, Branched-Chain Amino Acid Transaminase 1; BCAT2, Branched-Chain Amino Acid Transaminase 2; SHMT2, Serine Hydroxymethyltransferase 2; PHGDH, Phosphoglycerate Dehydrogenase; IDO1, Indoleamine 2,3-Dioxygenase 1; TDO2, Tryptophan 2,3-Dioxygenase 2; ARG1, Arginase 1; ARG2, Arginase 2; ASS1, Argininosuccinate Synthetase 1; ROS, Reactive Oxygen Species; OS, Overall Survival; PFS, Progression-Free Survival.

GC is an aggressive malignancy with a poor prognosis, and traditional therapeutic approaches such as surgery, neoadjuvant radiotherapy, and chemotherapy have inherent limitations and often fail to address the fundamental drivers of tumor progression. Thus, exploring innovative therapeutic strategies beyond conventional methods is imperative. Amino acids are essential for various vital biological processes, including protein synthesis, purine and pyrimidine biosynthesis, energy production, and redox balance maintenance. GC cells exhibit a high demand for amino acids, and alterations in amino acid levels can reflect the metabolic state of GC cells; additionally, key proteins or enzymes involved in amino acid metabolism hold potential as diagnostic markers and therapeutic targets for GC. Accumulating evidence indicates that reprogramming amino acid metabolism is crucial for regulating anti-tumor immunity, and this review has summarized the roles and mechanisms of catalytic enzymes related to amino acid metabolism in GC. Although conclusive evidence linking amino acid metabolism to GC initiation and progression is still lacking, metabolomic studies have identified significant differences in amino acid levels in tissues, serum, or urine between GC patients and healthy individuals, which may provide new insights for GC-related amino acid metabolism research. In the future, the clinical translation of amino acid metabolism-targeted therapy needs to focus on solving core technical bottlenecks, screen reliable biomarkers through multi-omics technology, optimize combination therapy regimens, and clarify drug resistance mechanisms. These efforts will lay the foundation for the development of novel anti-tumor drugs and therapeutic strategies, thereby promoting the advancement of precise metabolic therapy for GC and improving patient outcomes.

## Data Availability

Not applicable.

## References

[1] Sundar R , Nakayama I , Markar SR , Shitara K , van Laarhoven HWM , Janjigian YY , et al. Gastric cancer. Lancet. 2025; 405( 10494): 2087– 102. doi:10.1016/S0140-6736(25)00052-2. 40319897

[2] Chen Y , Jia K , Xie Y , Yuan J , Liu D , Jiang L , et al. The current landscape of gastric cancer and gastroesophageal junction cancer diagnosis and treatment in China: A comprehensive nationwide cohort analysis. J Hematol Oncol. 2025; 18( 1): 42. doi:10.1186/s13045-025-01698-y. 40234884 PMC12001465

[3] Li J , Wu Z , Lin R . Impact of *Helicobacter pylori* on immunotherapy in gastric cancer. J Immunother Cancer. 2024; 12( 10): e010354. doi:10.1136/jitc-2024-010354. 39438116 PMC11499768

[4] Pradhan SP , Gadnayak A , Pradhan SK , Epari V . Epidemiology and prevention of gastric cancer: A comprehensive review. Semin Oncol. 2025; 52( 3): 152341. doi:10.1016/j.seminoncol.2025.152341. 40305929

[5] Mithany RH , Shahid MH , Manasseh M , Saeed MT , Aslam S , Mohamed MS , et al. Gastric cancer: A comprehensive literature review. Cureus. 2024; 16( 3): e55902. doi:10.7759/cureus.55902. 38595903 PMC11003650

[6] Wang J , He Y , Hu F , Hu C , Sun Y , Yang K , et al. Metabolic reprogramming of immune cells in the tumor microenvironment. Int J Mol Sci. 2024; 25( 22): 12223. doi:10.3390/ijms252212223. 39596288 PMC11594648

[7] Zhao X , Li K , Chen M , Liu L . Metabolic codependencies in the tumor microenvironment and gastric cancer: Difficulties and opportunities. Biomed Pharmacother. 2023; 162: 114601. doi:10.1016/j.biopha.2023.114601. 36989719

[8] Khan MA , Zubair H , Anand S , Srivastava SK , Singh S , Singh AP . Dysregulation of metabolic enzymes in tumor and stromal cells: Role in oncogenesis and therapeutic opportunities. Cancer Lett. 2020; 473: 176– 85. doi:10.1016/j.canlet.2020.01.003. 31923436 PMC7067140

[9] Wu X , Tan X , Bao Y , Yan W , Zhang Y . Landscape of metabolic alterations and treatment strategies in breast cancer. Genes Dis. 2025; 12( 5): 101521. doi:10.1016/j.gendis.2025.101521. 40548063 PMC12179608

[10] Tang C , Corredeira P , Casimiro S , Shi Q , Han Q , Sukdao W , et al. Immunodiagnostic plasma amino acid residue biomarkers detect cancer early and predict treatment response. Nat Commun. 2025; 16: 6474. doi:10.1038/s41467-025-61685-2. 40659634 PMC12260101

[11] Shang Z , Ma Z , Wu E , Chen X , Tuo B , Li T , et al. Effect of metabolic reprogramming on the immune microenvironment in gastric cancer. Biomed Pharmacother. 2024; 170: 116030. doi:10.1016/j.biopha.2023.116030. 38128177

[12] Fan S , Guo J , Nie H , Xiong H , Xia Y . Aberrant energy metabolism in tumors and potential therapeutic targets. Genes Chromosomes Cancer. 2024; 63( 11): e70008. doi:10.1002/gcc.70008. 39584783 PMC11587691

[13] Cortes Ballen AI , Amosu M , Ravinder S , Chan J , Derin E , Slika H , et al. Metabolic reprogramming in glioblastoma multiforme: A review of pathways and therapeutic targets. Cells. 2024; 13( 18): 1574. doi:10.3390/cells13181574. 39329757 PMC11430559

[14] Shan Z , Liu Y . Harnessing glycolysis in gastric cancer: Molecular targets, therapeutic strategies, and clinical horizons. Front Immunol. 2025; 16: 1628937. doi:10.3389/fimmu.2025.1628937. 40977684 PMC12443842

[15] Zhang H , Ye W , Zeng L , Wang L , Gui L . Amino acid metabolism-related model for prognosis and immunity in gastric cancer. Amino Acids. 2026; 58( 1): 18. doi:10.1007/s00726-026-03507-3. 41784817 PMC12979369

[16] Feng T , Xie F , Lyu Y , Yu P , Chen B , Yu J , et al. The arginine metabolism and its deprivation in cancer therapy. Cancer Lett. 2025; 620: 217680. doi:10.1016/j.canlet.2025.217680. 40157492

[17] Bednarz-Misa I , Fleszar MG , Fortuna P , Lewandowski Ł , Mierzchała-Pasierb M , Diakowska D , et al. Altered L-arginine metabolic pathways in gastric cancer: Potential therapeutic targets and biomarkers. Biomolecules. 2021; 11( 8): 1086. doi:10.3390/biom11081086. 34439753 PMC8395015

[18] Zhu Y , Zhou Z , Du X , Lin X , Liang ZM , Chen S , et al. Cancer cell-derived arginine fuels polyamine biosynthesis in tumor-associated macrophages to promote immune evasion. Cancer Cell. 2025; 43( 6): 1045– 60. doi:10.1016/j.ccell.2025.03.015. 40185095

[19] Ohrt MM , Ing NH . Supplementary L-arginine can enhance reproductive parameters and outcomes in large mammals. Front Vet Sci. 2026; 12: 1740399. doi:10.3389/fvets.2025.1740399. 41602621 PMC12832522

[20] Abu-Soud HM , Camp OG , Ramadoss J , Chatzicharalampous C , Kofinas G , Kofinas JD . Regulation of nitric oxide generation and consumption. Int J Biol Sci. 2025; 21( 3): 1097– 109. doi:10.7150/ijbs.105016. 39897032 PMC11781162

[21] Gao D , Asghar S , Hu R , Chen S , Niu R , Liu J , et al. Recent advances in diverse nanosystems for nitric oxide delivery in cancer therapy. Acta Pharm Sin B. 2023; 13( 4): 1498– 521. doi:10.1016/j.apsb.2022.11.016. 37139410 PMC10149905

[22] Canè S , Geiger R , Bronte V . The roles of arginases and arginine in immunity. Nat Rev Immunol. 2025; 25( 4): 266– 84. doi:10.1038/s41577-024-01098-2. 39420221

[23] Han J , Gong S , Bian X , Qian Y , Wang G , Li N , et al. Polarity-regulated derivatization-assisted LC-MS method for amino-containing metabolites profiling in gastric cancer. J Pharm Anal. 2023; 13( 11): 1353– 64. doi:10.1016/j.jpha.2023.06.009. 38174119 PMC10759254

[24] Dash S , Firmanty P , Chomczyk M , Mohanty V , Ma W , Baran N . Targeting MDSCs in cancer: Emerging immunotherapeutic and metabolic strategies. Front Immunol. 2026; 17: 1749965. doi:10.3389/fimmu.2026.1749965. 41836430 PMC12982108

[25] Martinenaite E , Lecoq I , Aaboe-Jørgensen M , Ahmad SM , Perez-Penco M , Glöckner HJ , et al. Arginase-1-specific T cells target and modulate tumor-associated macrophages. J Immunother Cancer. 2025; 13( 1): e009930. doi:10.1136/jitc-2024-009930. 39880485 PMC11781113

[26] Ren W , Zhang X , Li W , Feng Q , Feng H , Tong Y , et al. Circulating and tumor-infiltrating arginase 1-expressing cells in gastric adenocarcinoma patients were mainly immature and monocytic Myeloid-derived suppressor cells. Sci Rep. 2020; 10( 1): 8056. doi:10.1038/s41598-020-64841-4. 32415175 PMC7229115

[27] Tishler H , Ziman S , Cheng K , Wang K , Sanghvi N , Adler L , et al. Inhibition of tumor microenvironment-driven JAK-STAT signaling enhances response to arginine deprivation therapy in triple-negative breast cancer. Cells. 2025; 15( 1): 25. doi:10.3390/cells15010025. 41511309 PMC12785028

[28] Hajaj E , Pozzi S , Erez A . From the inside out: Exposing the roles of urea cycle enzymes in tumors and their micro and macro environments. Cold Spring Harb Perspect Med. 2024; 14( 4): a041538. doi:10.1101/cshperspect.a041538. 37696657 PMC10982720

[29] Feng X , Ji Z , Yang G . ASS1 regulates immune microenvironment via CXCL8 signaling in ovarian cancer. Biochem Biophys Res Commun. 2022; 631: 86– 92. doi:10.1016/j.bbrc.2022.08.045. 36182868

[30] Bai D , Zhou Y , Jing L , Guo C , Yang Q . Arginine metabolism in cancer biology and immunotherapy. Immune Netw. 2025; 25( 4): e30. doi:10.4110/in.2025.25.e30. 40917794 PMC12411109

[31] Poeggeler B , Singh SK , Pappolla MA . Tryptophan in nutrition and health. Int J Mol Sci. 2022; 23( 10): 5455. doi:10.3390/ijms23105455. 35628285 PMC9146092

[32] Stone TW , Williams RO . Modulation of T cells by tryptophan metabolites in the kynurenine pathway. Trends Pharmacol Sci. 2023; 44( 7): 442– 56. doi:10.1016/j.tips.2023.04.006. 37248103

[33] Seo SK , Kwon B . Immune regulation through tryptophan metabolism. Exp Mol Med. 2023; 55( 7): 1371– 9. doi:10.1038/s12276-023-01028-7. 37394584 PMC10394086

[34] Lu Z , Zhang C , Zhang J , Su W , Wang G , Wang Z . The kynurenine pathway and indole pathway in tryptophan metabolism influence tumor progression. Cancer Med. 2025; 14( 6): e70703. doi:10.1002/cam4.70703. 40103267 PMC11919716

[35] Zheng Y , Yao Y , Ge T , Ge S , Jia R , Song X , et al. Amino acid metabolism reprogramming: Shedding new light on T cell anti-tumor immunity. J Exp Clin Cancer Res. 2023; 42( 1): 291. doi:10.1186/s13046-023-02845-4. 37924140 PMC10623764

[36] Wu Z , Wang H , Zheng Z , Lin Y , Bian L , Geng H , et al. IDO1 inhibition enhances CLDN18.2-CAR-T cell therapy in gastrointestinal cancers by overcoming kynurenine-mediated metabolic suppression in the tumor microenvironment. J Transl Med. 2025; 23( 1): 275. doi:10.1186/s12967-025-06276-x. 40045363 PMC11884131

[37] Ning W , Huang S . Spatially resolved tryptophan-kynurenine niches in HNSCC: Immunometabolic microdomains and therapeutic implications. Front Immunol. 2026; 17: 1756010. doi:10.3389/fimmu.2026.1756010. 41685324 PMC12891070

[38] Azimnasab-sorkhabi P , Soltani-asl M , Yoshinaga TT , Zaidan Dagli ML , de Oliveira Massoco C , Kfoury Junior JR . Indoleamine-2, 3 dioxygenase: A fate-changer of the tumor microenvironment. Mol Biol Rep. 2023; 50( 7): 6133– 45. doi:10.1007/s11033-023-08469-3. 37217614 PMC10202066

[39] Chen H , Zheng Q , Jiang Y , Lin L , Yang Y . IDO1 expression and CD8^+^ T-cell levels are useful prognostic biomarkers in preoperative gastric cancer specimens before neoadjuvant chemotherapy. Appl Immunohistochem Mol Morphol. 2025; 33( 1): 1– 9. doi:10.1097/PAI.0000000000001238. 39636312

[40] Xu X , Yuan H , Lv Q , Wu Z , Fan W , Liu J . Indoleamine 2, 3-dioxygenase regulates the differentiation of T lymphocytes to promote the growth of gastric cancer cells through the PI3K/Akt/mTOR pathway. Cell Biochem Biophys. 2025; 83( 2): 2289– 99. doi:10.1007/s12013-024-01641-x. 39695014 PMC12089202

[41] Cui JX , Xu XH , He T , Liu JJ , Xie TY , Tian W , et al. L-kynurenine induces NK cell loss in gastric cancer microenvironment via promoting ferroptosis. J Exp Clin Cancer Res. 2023; 42( 1): 52. doi:10.1186/s13046-023-02629-w. 36855135 PMC9976385

[42] Wang Z , Xie X , Xue Y , Chen Y . Tryptophan-2, 3-dioxygenase as a therapeutic target in digestive system diseases. Biology. 2025; 14( 3): 295. doi:10.3390/biology14030295. 40136551 PMC11939885

[43] Pham QT , Taniyama D , Akabane S , Takashima T , Maruyama R , Sekino Y , et al. Essential roles of TDO2 in gastric cancer: TDO2 is associated with cancer progression, patient survival, PD-L1 expression, and cancer stem cells. Pathobiology. 2023; 90( 1): 44– 55. doi:10.1159/000523750. 35679834

[44] Perrot-Applanat M , Pimpie C , Vacher S , Bieche I , Pocard M , Baud V . Differential expression of genes involved in metabolism and immune response in diffuse and intestinal gastric cancers, a pilot ptudy. Biomedicines. 2022; 10( 2): 240. doi:10.3390/biomedicines10020240. 35203450 PMC8869420

[45] Tu RH , Wu SZ , Huang ZN , Zhong Q , Ye YH , Zheng CH , et al. Neurotransmitter receptor HTR2B regulates lipid metabolism to inhibit ferroptosis in gastric cancer. Cancer Res. 2023; 83( 23): 3868– 85. doi:10.1158/0008-5472.CAN-23-1012. 38037454

[46] Zhang X , Zhao Y , Chen X . Collagen extracellular matrix promotes gastric cancer immune evasion by activating IL4I1-AHR signaling. Transl Oncol. 2024; 49: 102113. doi:10.1016/j.tranon.2024.102113. 39216468 PMC11402615

[47] Venkateswaran N , Garcia R , Lafita-Navarro MC , Hao YH , Perez-Castro L , Nogueira PAS , et al. Tryptophan fuels MYC-dependent liver tumorigenesis through indole 3-pyruvate synthesis. Nat Commun. 2024; 15: 4266. doi:10.1038/s41467-024-47868-3. 38769298 PMC11106337

[48] Zeitler L , Murray PJ . IL4i1 and IDO1: Oxidases that control a tryptophan metabolic nexus in cancer. J Biol Chem. 2023; 299( 6): 104827. doi:10.1016/j.jbc.2023.104827. 37196768 PMC10318530

[49] Jin J , Byun JK , Choi YK , Park KG . Targeting glutamine metabolism as a therapeutic strategy for cancer. Exp Mol Med. 2023; 55( 4): 706– 15. doi:10.1038/s12276-023-00971-9. 37009798 PMC10167356

[50] Ma G , Zhang Z , Li P , Zhang Z , Zeng M , Liang Z , et al. Reprogramming of glutamine metabolism and its impact on immune response in the tumor microenvironment. Cell Commun Signal. 2022; 20( 1): 114. doi:10.1186/s12964-022-00909-0. 35897036 PMC9327201

[51] Ni Q , Yu J , Niu Y , Han Z , Hu B , Wang Y , et al. Single-cell transcriptomic data reveal the cellular heterogeneity of glutamine metabolism in gastric premalignant lesions and early gastric cancer. Acta Biochim Biophys Sin. 2025; 57( 10): 1670– 83. doi:10.3724/abbs.2025061. 40264416 PMC12616717

[52] Nan D , Yao W , Huang L , Liu R , Chen X , Xia W , et al. Glutamine and cancer: Metabolism, immune microenvironment, and therapeutic targets. Cell Commun Signal. 2025; 23( 1): 45. doi:10.1186/s12964-024-02018-6. 39856712 PMC11760113

[53] De Los Santos-Jiménez J , Campos-Sandoval JA , Alonso FJ , Márquez J , Matés JM . GLS and GLS2 glutaminase isoenzymes in the antioxidant system of cancer cells. Antioxidants. 2024; 13( 6): 745. doi:10.3390/antiox13060745. 38929183 PMC11200642

[54] Li S , Zeng H , Fan J , Wang F , Xu C , Li Y , et al. Glutamine metabolism in breast cancer and possible therapeutic targets. Biochem Pharmacol. 2023; 210: 115464. doi:10.1016/j.bcp.2023.115464. 36849062

[55] Li D , Cao D , Zhang Y , Yu X , Wu Y , Jia Z , et al. Integrative pan-cancer analysis and experiment validation identified GLS as a biomarker in tumor progression, prognosis, immune microenvironment, and immunotherapy. Sci Rep. 2025; 15: 525. doi:10.1038/s41598-024-84916-w. 39747578 PMC11696030

[56] Yang J , Chen F , Lang L , Yang F , Fu Z , Martinez J , et al. Therapeutic targeting of the GLS1-c-myc positive feedback loop suppresses glutaminolysis and inhibits progression of head and neck cancer. Cancer Res. 2024; 84( 19): 3223– 34. doi:10.1158/0008-5472.CAN-24-0254. 39024547 PMC11444885

[57] Dong M , Song Z , Lu X , Lu M , Zhong C . CENPF promotes gastric cancer proliferation through c-myc-mediated GLS1 upregulation and glutamine metabolism. Oncol Res. 2026; 34( 3): 22. doi:10.32604/or.2026.068508. PMC12963658 41799529

[58] Liu J , Hong S , Gu K . A pancancer analysis reveals the oncogenic role of glutaminase 1 (GLS1) in tumor metabolism and immune evasion: A bioinformatics analysis. Discov Oncol. 2025; 16( 1): 2156. doi:10.1007/s12672-025-03779-3. 41284103 PMC12644386

[59] Jamshidi-Parsian A , Jenkins SV , Tran A , Bragg A , Davis R , Griffin C , et al. CB-839 induces reversible dormancy in lung tumor-cells. Eur J Pharmacol. 2024; 982: 176912. doi:10.1016/j.ejphar.2024.176912. 39159716 PMC12702564

[60] Buczkowska J , Szeliga M . Two faces of glutaminase GLS2 in carcinogenesis. Cancers. 2023; 15( 23): 5566. doi:10.3390/cancers15235566. 38067269 PMC10705333

[61] Suzuki S , Venkatesh D , Kanda H , Nakayama A , Hosokawa H , Lee E , et al. GLS2 is a tumor suppressor and a regulator of ferroptosis in hepatocellular carcinoma. Cancer Res. 2022; 82( 18): 3209– 22. doi:10.1158/0008-5472.CAN-21-3914. 35895807 PMC11057045

[62] Wang X , Tan X , Zhang J , Wu J , Shi H . The emerging roles of MAPK-AMPK in ferroptosis regulatory network. Cell Commun Signal. 2023; 21( 1): 200. doi:10.1186/s12964-023-01170-9. 37580745 PMC10424420

[63] Cyriac R , Lee K . Glutaminase inhibition as potential cancer therapeutics: Current status and future applications. J Enzyme Inhib Med Chem. 2024; 39( 1): 2290911. doi:10.1080/14756366.2023.2290911. 38078371 PMC11721875

[64] Zheng H , Zhang X , Li C , Wang D , Shen Y , Lu J , et al. BCAA mediated microbiota-liver-heart crosstalk regulates diabetic cardiomyopathy via FGF21. Microbiome. 2024; 12( 1): 157. doi:10.1186/s40168-024-01872-3. 39182099 PMC11344321

[65] Wang L , Shi F , Cao Y , Xie L . Multiple roles of branched-chain amino acid metabolism in tumour progression. J Biomed Sci. 2025; 32( 1): 41. doi:10.1186/s12929-025-01132-y. 40205401 PMC11983764

[66] Xu H , Wang X , Xu X , Liu L , Zhang Y , Yan X , et al. Association of plasma branched-chain amino acid with multiple cancers: A Mendelian randomization analysis. Clin Nutr. 2023; 42( 12): 2493– 502. doi:10.1016/j.clnu.2023.10.019. 37922693

[67] Shu X , Zhan PP , Sun LX , Yu L , Liu J , Sun LC , et al. BCAT1 activates PI3K/AKT/mTOR pathway and contributes to the angiogenesis and tumorigenicity of gastric cancer. Front Cell Dev Biol. 2021; 9: 659260. doi:10.3389/fcell.2021.659260. 34164393 PMC8215359

[68] Liu R , Liu J , Wu P , Yi H , Zhang B , Huang W . Flotillin-2 promotes cell proliferation via activating the c-Myc/BCAT1 axis by suppressing miR-33b-5p in nasopharyngeal carcinoma. Aging. 2021; 13( 6): 8078– 94. doi:10.18632/aging.202726. 33744853 PMC8034900

[69] Kubickova A , De Sanctis JB , Hajduch M . Isoform-directed control of c-myc functions: Understanding the balance from proliferation to growth arrest. Int J Mol Sci. 2023; 24( 24): 17524. doi:10.3390/ijms242417524. 38139353 PMC10743581

[70] Mi Y , Shan H , Wang B , Tang H , Jia J , Liu X , et al. Genipin inhibits proliferation of gastric cancer cells by inducing ferroptosis: An integrated study of network pharmacology and bioinformatics study. Med Oncol. 2024; 41( 2): 46. doi:10.1007/s12032-023-02283-4. 38175425

[71] Cao Q , Fan J , Zou J , Wang W . Multi-omics analysis identifies BCAT2 as a potential pan-cancer biomarker for tumor progression and immune microenvironment modulation. Sci Rep. 2024; 14( 1): 23371. doi:10.1038/s41598-024-74441-1. 39375392 PMC11458862

[72] Jiang Y , Tao Q , Qiao X , Yang Y , Peng C , Han M , et al. Targeting amino acid metabolism to inhibit gastric cancer progression and promote anti-tumor immunity: A review. Front Immunol. 2025; 16: 1508730. doi:10.3389/fimmu.2025.1508730. 40018041 PMC11864927

[73] Shan Y , Liu D , Li Y , Wu C , Ye Y . The expression and clinical significance of serine hydroxymethyltransferase2 in gastric cancer. PeerJ. 2024; 12: e16594. doi:10.7717/peerj.16594. 38188143 PMC10771762

[74] Shu M , Liu Y , Wang J . Protein post-translational modifications in serine synthetic pathway: Functions and molecular mechanisms. Cell Commun Signal. 2025; 23( 1): 311. doi:10.1186/s12964-025-02327-4. 40598535 PMC12211383

[75] Geeraerts SL , Heylen E , De Keersmaecker K , Kampen KR . The ins and outs of serine and *Glycine* metabolism in cancer. Nat Metab. 2021; 3( 2): 131– 41. doi:10.1038/s42255-020-00329-9. 33510397

[76] Su P , Yang Y , Zheng H . The “serine code” of metabolic reprogramming: Multidimensional roles of the serine synthesis pathway in tumors and novel breakthroughs for targeted therapy. Front Immunol. 2026; 17: 1779543. doi:10.3389/fimmu.2026.1779543. 41816347 PMC12971912

[77] Lee CM , Hwang Y , Kim M , Park YC , Kim H , Fang S . PHGDH: A novel therapeutic target in cancer. Exp Mol Med. 2024; 56( 7): 1513– 22. doi:10.1038/s12276-024-01268-1. 38945960 PMC11297271

[78] Yoon BK , Kim H , Oh TG , Oh SK , Jo S , Kim M , et al. PHGDH preserves one-carbon cycle to confer metabolic plasticity in chemoresistant gastric cancer during nutrient stress. Proc Natl Acad Sci U S A. 2023; 120( 21): e2217826120. doi:10.1073/pnas.2217826120. 37192160 PMC10214193

[79] Zhu S , Liu Y , Chen H , Zhu X , Liu X , Xu K , et al. Mechanism and therapeutic progress of one-carbon metabolic key enzyme: Serine hydroxymethyltransferase 2 in cancer. Clin Med Insights Oncol. 2025; 19: 11795549251331755. doi:10.1177/11795549251331755. 40337354 PMC12056339

[80] Li J , Bo Y , Ding B , Wang L . Understanding the regulatory role of USP32 and SHMT2 in the progression of gastric cancer. Cell J. 2023; 25( 4): 222– 8. doi:10.22074/cellj.2022.557384.1046. 37210642 PMC10201363

[81] Wang W , Wang M , Du T , Hou Z , You S , Zhang S , et al. SHMT2 promotes gastric cancer development through regulation of HIF1α/VEGF/STAT3 signaling. Int J Mol Sci. 2023; 24( 8): 7150. doi:10.3390/ijms24087150. 37108312 PMC10138966

[82] Wang M , Yu K , Meng F , Wang H , Li Y . NEK8 promotes the progression of gastric cancer by reprogramming asparagine metabolism. Mol Med. 2025; 31( 1): 3. doi:10.1186/s10020-024-01062-9. 39762761 PMC11702068

[83] Ge M , Wang L , Zheng B , Zhan L , Cui L , Wang H , et al. Targeting asparagine potentiates anti-PD-L1 immunotherapy in gastric cancer by enhancing CD8^+^ T cell anti-tumor response. Front Immunol. 2025; 16: 1626581. doi:10.3389/fimmu.2025.1626581. 41229436 PMC12603620

[84] Zhang J , Chen M , Yang Y , Liu Z , Guo W , Xiang P , et al. Amino acid metabolic reprogramming in the tumor microenvironment and its implication for cancer therapy. J Cell Physiol. 2024; 239( 11): e31349. doi:10.1002/jcp.31349. 38946173

[85] Guo Z , Xiang Z , Su W , Lv B , Zhao Q , Zhang W , et al. Metabolic regulation of amino acids provides an important basis for individualized nutritional therapy for patients with gastric cancer during the perioperative period. World J Surg Oncol. 2025; 23( 1): 89. doi:10.1186/s12957-025-03729-x. 40087750 PMC11907831

[86] Mao L , Wang L , Lyu Y , Zhuang Q , Li Z , Zhang J , et al. Branch chain amino acid metabolism promotes brain metastasis of NSCLC through EMT occurrence by regulating ALKBH5 activity. Int J Biol Sci. 2024; 20( 9): 3285– 301. doi:10.7150/ijbs.85672. 38993559 PMC11234221

[87] Peng R , Dong Y , Zheng M , Kang H , Wang P , Zhu M , et al. IL-17 promotes osteoclast-induced bone loss by regulating glutamine-dependent energy metabolism. Cell Death Dis. 2024; 15( 2): 111. doi:10.1038/s41419-024-06475-2. 38316760 PMC10844210

[88] Qiu Y , Stamatatos OT , Hu Q , Ruiter Swain J , Russo S , Sann A , et al. The unique catalytic properties of PSAT1 mediate metabolic adaptation to glutamine blockade. Nat Metab. 2024; 6( 8): 1529– 48. doi:10.1038/s42255-024-01104-w. 39192144 PMC11490312

[89] Liu D , Yi H , Yi C . Redox-amino acid metabolic crosstalk in ovarian cancer stem cells: Integrating metabolic reprogramming, signaling, and the tumor microenvironment. Antioxidants. 2025; 14( 12): 1413. doi:10.3390/antiox14121413. 41462613 PMC12729957

[90] Kang Z , Wu B , Zhang L , Liang X , Guo D , Yuan S , et al. Metabolic regulation by biomaterials in osteoblast. Front Bioeng Biotechnol. 2023; 11: 1184463. doi:10.3389/fbioe.2023.1184463. 37324445 PMC10265685

[91] Nong X , Zhang C , Wang J , Ding P , Ji G , Wu T . The mechanism of branched-chain amino acid transferases in different diseases: Research progress and future prospects. Front Oncol. 2022; 12: 988290. doi:10.3389/fonc.2022.988290. 36119495 PMC9478667

[92] Sun Y , Shen Y , Yan Y , Luo W , Liu C , Tang J . GLS1 promotes lipid metabolism in hepatocellular carcinoma by regulating the PI3K/AKT/mTORC1 signaling pathway through SREBP-1. Am J Transl Res. 2025; 17( 4): 2527– 40. doi:10.62347/ZTGP5030. 40385002 PMC12082496

[93] Field GC , Pavlyk I , Szlosarek PW . Bench-to-bedside studies of arginine deprivation in cancer. Molecules. 2023; 28( 5): 2150. doi:10.3390/molecules28052150. 36903394 PMC10005060

[94] Chu YD , Lai MW , Yeh CT . Unlocking the potential of arginine deprivation therapy: Recent breakthroughs and promising future for cancer treatment. Int J Mol Sci. 2023; 24( 13): 10668. doi:10.3390/ijms241310668. 37445845 PMC10341449

[95] Carpentier J , Pavlyk I , Mukherjee U , Hall PE , Szlosarek PW . Arginine deprivation in SCLC: Mechanisms and perspectives for therapy. Lung Cancer Targets Ther. 2022; 13: 53– 66. doi:10.2147/LCTT.S335117. PMC9462517 36091646

[96] Shi LY , Wang YY , Jing Y , Xu MH , Zhu ZT , Wang QJ . Abnormal arginine metabolism is associated with prognosis in patients of gastric cancer. Transl Cancer Res. 2021; 10( 5): 2451– 69. doi:10.21037/tcr-21-794. 35116560 PMC8797619

[97] Ming Z , Luo T , Zou Z , Luo W , Hu X , Chen L , et al. ASS1 inhibits liver cancer by promoting CAD ubiquitination and reversing the urea cycle and pyrimidine synthesis imbalance. Hepatol Commun. 2025; 9( 9): e0769. doi:10.1097/HC9.0000000000000769. 40824254 PMC12363443

[98] Luo W , Zou Z , Nie Y , Luo J , Ming Z , Hu X , et al. ASS1 inhibits triple-negative breast cancer by regulating PHGDH stability and *de novo* serine synthesis. Cell Death Dis. 2024; 15: 319. doi:10.1038/s41419-024-06672-z. 38710705 PMC11074131

[99] Zhao R , He B , Huang L , Wu Y , Liu T , Liu J , et al. Targeting AQP5-mediated arginine deprivation in gastric cancer stem cells restores NK cell anti-tumor immunity. Cell Rep Med. 2025; 6( 9): 102333. doi:10.1016/j.xcrm.2025.102333. 40961922 PMC12490235

[100] Chen CL , Hsu SC , Ann DK , Yen Y , Kung HJ . Arginine signaling and cancer metabolism. Cancers. 2021; 13( 14): 3541. doi:10.3390/cancers13143541. 34298755 PMC8306961

[101] Gao M , Yu W , Xi Z , Zhang Z , Fan X , Wang X . Recent update on the discovery of indoleamine-2, 3-dioxygenase 1 inhibitors targeting cancer immunotherapy. Eur J Med Chem. 2025; 298: 118017. doi:10.1016/j.ejmech.2025.118017. 40752342

[102] Liao H , Ma Q , Chen L , Guo W , Feng K , Bao Y , et al. Machine learning analysis of CD4^+^ T cell gene expression in diverse diseases: Insights from cancer, metabolic, respiratory, and digestive disorders. Cancer Genet. 2025; 290–291: 56– 60. doi:10.1016/j.cancergen.2024.12.004. 39729927

[103] Al-Zoubi RM , Elaarag M , Al-Qudimat AR , Al-Hurani EA , Fares ZE , Farhan A , et al. IDO and TDO inhibitors in cancer immunotherapy: Mechanisms, clinical development, and future directions. Front Pharmacol. 2025; 16: 1632446. doi:10.3389/fphar.2025.1632446. 41035912 PMC12481718

[104] Li M , Wang T , Zhang Z , Dongye Y . Metabolic reprogramming in the post-metastatic tumor microenvironment: Multi-omics insights into determinants of immunotherapy response. Front Immunol. 2026; 16: 1742855. doi:10.3389/fimmu.2025.1742855. 41562065 PMC12813170

[105] Yan J , Chen D , Ye Z , Zhu X , Li X , Jiao H , et al. Molecular mechanisms and therapeutic significance of Tryptophan Metabolism and signaling in cancer. Mol Cancer. 2024; 23( 1): 241. doi:10.1186/s12943-024-02164-y. 39472902 PMC11523861

[106] Cinar I , Dincer B , Cadirci E , Kara S , Yildirgan MI , Halici Z , et al. Antagonism of 5-HT7 receptors as a promising target for gastric cancer via apoptotic pathway. J Biochem Mol Toxicol. 2025; 39( 6): e70326. doi:10.1002/jbt.70326. 40488271 PMC12147198

[107] Vagaggini C , D’Ursi P , Poggialini F , Fossa P , Francesconi V , Trombetti G , et al. Deciphering the landscape of allosteric glutaminase 1 inhibitors as anticancer agents. Bioorganic Chem. 2025; 161: 108523. doi:10.1016/j.bioorg.2025.108523. 40311238

[108] Gorgoglione R , Impedovo V , Riley CL , Fratantonio D , Tiziani S , Palmieri L , et al. Glutamine-derived aspartate biosynthesis in cancer cells: Role of mitochondrial transporters and new therapeutic perspectives. Cancers. 2022; 14( 1): 245. doi:10.3390/cancers14010245. 35008407 PMC8750728

[109] Zhang M , Lu N , Li HJ , Guo XY , Lu L , Guo Y . Inhibition of lncRNA NEAT1 induces dysfunction of fibroblast-like synoviocytes in rheumatoid arthritis via miRNA-338-3p-mediated regulation of glutamine metabolism. J Orthop Surg Res. 2022; 17( 1): 401. doi:10.1186/s13018-022-03295-y. 36050752 PMC9438172

[110] Chen T , Zhao L , Chen S , Zheng B , Chen H , Zeng T , et al. The curcumin analogue WZ35 affects glycolysis inhibition of gastric cancer cells through ROS-YAP-JNK pathway. Food Chem Toxicol. 2020; 137: 111131. doi:10.1016/j.fct.2020.111131. 31958483

[111] Zhang Y , Liu W , Feng W , Wang X , Lei T , Chen Z , et al. Identification of 14 differentially-expressed metabolism-related genes as potential targets of gastric cancer by integrated proteomics and transcriptomics. Front Cell Dev Biol. 2022; 10: 816249. doi:10.3389/fcell.2022.816249. 35265615 PMC8899292

[112] Kohli M , Poulogiannis G . Harnessing the power of metabolomics for precision oncology: Current advances and future directions. Cells. 2025; 14( 6): 402. doi:10.3390/cells14060402. 40136651 PMC11940876

[113] Niu F , Yu Y , Li Z , Ren Y , Li Z , Ye Q , et al. Arginase: An emerging and promising therapeutic target for cancer treatment. Biomed Pharmacother. 2022; 149: 112840. doi:10.1016/j.biopha.2022.112840. 35316752

[114] Menjivar RE , Nwosu ZC , Du W , Donahue KL , Hong HS , Espinoza C , et al. Arginase 1 is a key driver of immune suppression in pancreatic cancer. eLife. 2023; 12: e80721. doi:10.7554/eLife.80721. 36727849 PMC10260021

[115] Borek B , Nowicka J , Gzik A , Dziegielewski M , Jedrzejczak K , Brzezinska J , et al. Arginase 1/2 inhibitor OATD-02: From discovery to first-in-man setup in cancer immunotherapy. Mol Cancer Ther. 2023; 22( 7): 807– 17. doi:10.1158/1535-7163.MCT-22-0721. 36939275

[116] Marzęta-Assas P , Jacenik D , Zasłona Z . Pathophysiology of arginases in cancer and efforts in their pharmacological inhibition. Int J Mol Sci. 2024; 25( 18): 9782. doi:10.3390/ijms25189782. 39337272 PMC11431790

[117] Liu S , Sun H , Du Z , Lu S , Wang C , Zhang Y , et al. Metabolomics and proteomics reveal blocking argininosuccinate synthetase 1 alleviates colitis in mice. Nat Commun. 2025; 16: 6983. doi:10.1038/s41467-025-62217-8. 40739098 PMC12311138

[118] Chan PY , Phillips MM , Ellis S , Johnston A , Feng X , Arora A , et al. A Phase 1 study of ADI-PEG20 (pegargiminase) combined with cisplatin and pemetrexed in ASS1-negative metastatic uveal melanoma. Pigment Cell Melanoma Res. 2022; 35( 4): 461– 70. doi:10.1111/pcmr.13042. 35466524 PMC9322321

[119] Rossini S , Ambrosino S , Volpi C , Suvieri C , Pallotta MT , Belladonna ML , et al. Adverse pro-tumorigenic effects of IDO1 catalytic inhibitors mediated by the non-enzymatic function of IDO1 in tumor cells. Front Immunol. 2025; 16: 1680896. doi:10.3389/fimmu.2025.1680896. 41262240 PMC12623407

[120] Zhou Y , Tao Q , Luo C , Chen J , Chen G , Sun J . Epacadostat overcomes cetuximab resistance in colorectal cancer by targeting IDO-mediated tryptophan metabolism. Cancer Sci. 2025; 116( 6): 1715– 29. doi:10.1111/cas.70057. 40103010 PMC12127106

[121] Liu M , Wang X , Wang L , Ma X , Gong Z , Zhang S , et al. Targeting the IDO1 pathway in cancer: From bench to bedside. J Hematol Oncol. 2018; 11( 1): 100. doi:10.1186/s13045-018-0644-y. 30068361 PMC6090955

[122] Hu S , Lu H , Xie W , Wang D , Shan Z , Xing X , et al. TDO2^+^ myofibroblasts mediate immune suppression in malignant transformation of squamous cell carcinoma. J Clin Investig. 2022; 132( 19): e157649. doi:10.1172/JCI157649. 35972800 PMC9525123

[123] Li S , Zhang Y , Luo M , Zhou W , Chen Y , Wu D , et al. AR to GR switch modulates differential TDO2-Kyn-AhR signalling to promote the survival and recurrence of treatment-induced dormant cells in prostate cancer. Cell Discov. 2025; 11: 67. doi:10.1038/s41421-025-00817-w. 40759641 PMC12322048

[124] Bai C , Hua J , Meng D , Xu Y , Zhong B , Liu M , et al. Glutaminase-1 mediated glutaminolysis to glutathione synthesis maintains redox homeostasis and modulates ferroptosis sensitivity in cancer cells. Cell Prolif. 2025; 58( 11): e70036. doi:10.1111/cpr.70036. 40259435 PMC12584870

[125] Zhu L , Chang X , Zhang S , Bai X , Finko AV , Xu X , et al. Enhancing the affinity of novel GLS1 allosteric inhibitors by targeting key residue Lys320. Future Med Chem. 2023; 15( 15): 1393– 414. doi:10.4155/fmc-2023-0114. 37610850

[126] Zhang T , Cui Y , Wu Y , Meng J , Han L , Zhang J , et al. Mitochondrial GCN5L1 regulates glutaminase acetylation and hepatocellular carcinoma. Clin Transl Med. 2022; 12( 5): e852. doi:10.1002/ctm2.852. 35538890 PMC9091986

[127] Günther J , Hillig RC , Zimmermann K , Kaulfuss S , Lemos C , Nguyen D , et al. BAY-069, a novel (trifluoromethyl)pyrimidinedione-based BCAT1/2 inhibitor and chemical probe. J Med Chem. 2022; 65( 21): 14366– 90. doi:10.1021/acs.jmedchem.2c00441. 36261130 PMC9661481

[128] Zhang B , Peng H , Zhou M , Bao L , Wang C , Cai F , et al. Targeting BCAT1 combined with α-ketoglutarate triggers metabolic synthetic lethality in glioblastoma. Cancer Res. 2022; 82( 13): 2388– 402. doi:10.1158/0008-5472.CAN-21-3868. 35499760 PMC9256772

[129] Yuan Z , Li M , Tang Z . BCAT1 promotes cell proliferation, migration, and invasion via the PI3K-Akt signaling pathway in oral squamous cell carcinoma. Oral Dis. 2025; 31( 2): 364– 75. doi:10.1111/odi.15084. 39056279

[130] Wilke AC , Doebele C , Zindel A , Lee KS , Rieke SA , Ceribelli M , et al. SHMT2 inhibition disrupts the TCF3 transcriptional survival program in Burkitt lymphoma. Blood. 2022; 139( 4): 538– 53. doi:10.1182/blood.2021012081. 34624079 PMC8938936

[131] Sun T , Chen Y , Chen YX . Single-cell and bulk transcriptome analyses reveal elevated amino acid metabolism promoting tumor-directed immune evasion in colorectal cancer. Front Immunol. 2025; 16: 1575829. doi:10.3389/fimmu.2025.1575829. 40475762 PMC12137356

[132] Shi M , Huai Y , Deng T , Zhang C , Song J , Wang J , et al. SHMT2 is essential for mammalian preimplantation embryonic development through *de novo* biosynthesis of nucleotide metabolites. Mol Ther Nucleic Acids. 2025; 36( 2): 102499. doi:10.1016/j.omtn.2025.102499. 40171278 PMC11960634

[133] Zhang X , Sun M , Jiao Y , Lin B , Yang Q . PHGDH inhibitor CBR-5884 inhibits epithelial ovarian cancer progression via ROS/wnt/β-catenin pathway and plays a synergistic role with PARP inhibitor olaparib. Oxid Med Cell Longev. 2022; 2022: 9029544. doi:10.1155/2022/9029544. 36105480 PMC9467758

[134] Chen C , Zhu T , Liu X , Zhu D , Zhang Y , Wu S , et al. Identification of a novel PHGDH covalent inhibitor by chemical proteomics and phenotypic profiling. Acta Pharm Sin B. 2022; 12( 1): 246– 61. doi:10.1016/j.apsb.2021.06.008. 35127383 PMC8799887

[135] Cao XY , Li X , Wang F , Duan Y , Wu X , Lin GQ , et al. Identification of benzo [b] thiophene-1, 1-dioxide derivatives as novel PHGDH covalent inhibitors. Bioorganic Chem. 2024; 146: 107330. doi:10.1016/j.bioorg.2024.107330. 38579615

[136] Gao D , Tang S , Cen Y , Yuan L , Lan X , Li QH , et al. Discovery of novel drug-like PHGDH inhibitors to disrupt serine biosynthesis for cancer therapy. J Med Chem. 2023; 66( 1): 285– 305. doi:10.1021/acs.jmedchem.2c01202. 36594670

[137] Sadiqa A , Rasul A , Hassan M , Sultana S , Jabeen F . Identification of novel natural inhibitors to human 3-phosphoglycerate dehydrogenase (PHGDH) for cancer treatment. Molecules. 2022; 27( 18): 6108. doi:10.3390/molecules27186108. 36144843 PMC9501931

